# New Insights in Cardiac β-Adrenergic Signaling During Heart Failure and Aging

**DOI:** 10.3389/fphar.2018.00904

**Published:** 2018-08-10

**Authors:** Claudio de Lucia, Akito Eguchi, Walter J. Koch

**Affiliations:** Department of Pharmacology – Center for Translational Medicine, Lewis Katz School of Medicine, Temple University, Philadelphia, PA, United States

**Keywords:** beta-adrenergic receptors, heart failure, cardiac aging, GRK2, pharmacology, sympathetic nervous system, cardiovascular system

## Abstract

Heart failure (HF) has become increasingly common within the elderly population, decreasing their survival and overall quality of life. In fact, despite the improvements in treatment, many elderly people suffer from cardiac dysfunction (HF, valvular diseases, arrhythmias or hypertension-induced cardiac hypertrophy) that are much more common in an older fragile heart. Since β-adrenergic receptor (β-AR) signaling is abnormal in failing as well as aged hearts, this pathway is an effective diagnostic and therapeutic target. Both HF and aging are characterized by activation/hyperactivity of various neurohormonal pathways, the most important of which is the sympathetic nervous system (SNS). SNS hyperactivity is initially a compensatory mechanism to stimulate contractility and maintain cardiac output. Unfortunately, this chronic stimulation becomes detrimental and causes decreased cardiac function as well as reduced inotropic reserve due to a decrease in cardiac β-ARs responsiveness. Therapies which (e.g., β-blockers and physical activity) restore β-ARs responsiveness can ameliorate cardiac performance and outcomes during HF, particularly in older patients. In this review, we will discuss physiological β-adrenergic signaling and its alterations in both HF and aging as well as the potential clinical application of targeting β-adrenergic signaling in these disease processes.

## Introduction

Heart failure (HF) is a leading cause of morbidity and mortality in western countries. Particularly, HF prevalence has increased to 6.5 million in Americans ≥20 years of age (Benjamin et al., 2017). Over the last several years, there has been an increase in the incidence and prevalence of HF in the elderly, drastically impacting their survival rate and quality of life (Shakib and Clark, 2016; Ziaeian and Fonarow, 2016). HF represents the final common clinical event of numerous cardiovascular diseases (CVDs); coronary artery disease (CAD) followed by myocardial ischemia is the most common cause worldwide. It is extensively recognized that dyslipidemia, diabetes, hypertension, obesity, metabolic syndrome, and an inactive lifestyle are major risk factors for CAD and HF (Mozaffarian et al., 2016). Importantly, all these factors have a higher prevalence in the older population and age as well as age-related diseases, are a risk factor for CVDs (Niccoli and Partridge, 2012; North and Sinclair, 2012; de Lucia et al., 2013; Gacci et al., 2016). Undoubtedly, it is crucial to invest in therapies that are able to counteract the effects of HF on cardiac function and at the same time improve the alterations that are unique to the aging heart. In this regard, we will discuss the role of cardiac β-adrenergic signaling in HF and aging. During HF and aging, several neurohormonal mechanisms are activated in order to maintain cardiac output. The most important among these are the sympathetic nervous system (SNS) overdrive characterized by elevated circulating catecholamines (CAs) and Renin–Angiotensin–Aldosterone System hyperactivity (Mendzef and Slovinski, 2004; Conti et al., 2012). Interestingly, due to chronic stimulation by CAs, cardiac β-adrenergic receptor (β-AR) responsiveness is altered in both HF and aging, and treatments (e.g., β-blockers and physical activity) that improve their signaling can ameliorate cardiac performance and outcomes during HF, particularly in older aged patients (Baxter et al., 2002; Leosco et al., 2007; Femminella et al., 2013; Rengo et al., 2013; Ferrara et al., 2014). However, despite considerable advances in preventive medicine and pharmacological treatments, heart disease still represents a severe clinical, social, and economic burden (Ponikowski et al., 2014). Hence, it is important to develop new drugs that will be able to further blunt cardiac dysfunction in elderly people with CVDs. Targeting β-AR signaling seems to be a wise choice since it is crucial for cardiac function and its manipulation has already been shown to be effective. In this review, we will primarily discuss cardiac β-adrenergic signaling at the molecular level and then we will specifically examine its role during HF and physiological aging. Finally, we will provide a translational and clinical perspective as well as future directions.

## Cardiac β-Adrenergic Signaling

### β-Adrenergic Receptors

The SNS is one of two branches of the nervous system that is involved in the regulation of numerous homeostatic mechanisms including cardiac function (Lymperopoulos et al., 2013). The main function of the SNS is to stimulate the body’s fight-or-flight response. The two molecules that are utilized in SNS signaling are the CAs epinephrine (Epi), released by the adrenal medulla, and norepinephrine (NEpi), also released by the adrenal medulla (minor) as well as from sympathetic nerve endings (de Lucia et al., 2014). These mediators circulate throughout the body and act on adrenergic receptors such as those expressed in the heart, eliciting a positive inotropic response. In diseased states including HF, elevated CA levels can cause detrimental effects on the heart including promoting maladaptive cardiac hypertrophy and cell death.

**FIGURE 1:**
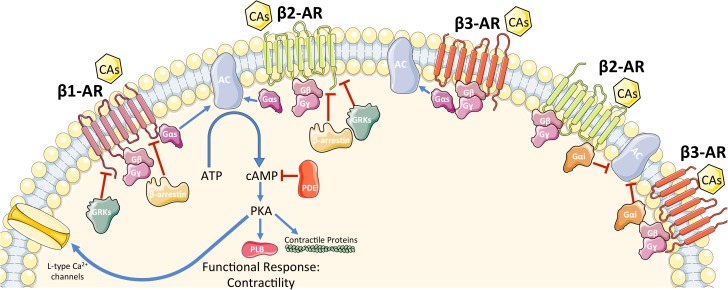
Schematic representation of β-AR signaling in cardiomyocytes. See main text for details. CAs, catecholamines; β-AR, β-adrenergic receptor; G-protein subunits: G_α_ (G_αs_ or G_αi_), G_β_, G_γ_; GRK2, G protein-coupled receptor kinase 2; AC, adenylyl cyclase; ATP, adenosine tri-phosphate; cAMP, cyclic adenosine mono-phosphate; PDE, phosphodiesterase; PKA, protein kinase A. A blue arrow is used when a stimulatory mechanism is involved while a red bar-headed line is used for an inhibitory mechanism.

β-Adrenergic receptors are members of the G protein-coupled receptor (GPCR) superfamily of receptors whose signaling plays a critical role in the regulation of the function and processes of the cardiovascular system. Presently, three subtypes of β-ARs have been characterized (β_1_-AR, β_2_-AR, β_3_-AR) with a fourth (β_4_-AR) remaining controversial (Gauthier et al., 1996; Zhu et al., 2011). Intracellular signaling of each β-AR has been summarized in **Figure 1**. The three subtypes have different affinities for different ligands, which allow variable activation of each subtype (Bristow et al., 1986; Lohse et al., 2003; Zhu et al., 2011). In the healthy human heart, there is approximately a 4:1 ratio of β_1_-AR to β_2_-AR, with minimal expression of β_3_-AR (Bristow et al., 1986; Moniotte et al., 2001). It has recently been confirmed that β_1_-ARs are present in all cardiomyocytes (Myagmar et al., 2017). Interestingly, Myagmar et al. (2017) found that β_2_-AR and β_3_-AR are frequently absent in myocytes (detected in only ≈5% of myocytes) but are abundant in non-myocyte cells (mainly endothelial cells in this study). On the other side, β_1_-AR is expressed at a low level in non-myocytes (Myagmar et al., 2017). Both β_1_-AR and β_2_-AR activation lead to increased inotropy, lusitropy, and chronotropy (Brodde and Michel, 1999); however, persistent β_2_-AR activation can lead to the reversal of these effects (Lefkowitz, 1998). β_3_-AR is similar to β_2_-AR in that regard, as it can have both stimulatory and inhibitory effects on the heart (Lefkowitz, 1998). β-ARs, like other GPCRs, consist of a seven-transmembrane-spanning receptor and are coupled to an intracellular heterotrimeric G-protein complex (Rockman et al., 2002). After agonist binding, the receptor undergoes a conformational change that induces an exchange of guanosine diphosphate (GDP) for guanosine triphosphate (GTP), causing dissociation of the now active G_α_ and G_βγ_ protein subunits. The activated G_α_ subunit then regulates the effector molecules downstream of the receptor. The identity of the downstream effectors is determined by the subtype of β-AR that is activated (Rockman et al., 2002). While all β-ARs are associated with the stimulatory G protein (G_αs_) activation, it is known that β_2_-AR and β_3_-AR can be coupled to inhibitory G protein according to studies which showed that their activity was pertussis toxin sensitive (G_αi_) (Kohout et al., 2001; Rockman et al., 2002). The activation of the G_αs_ subunit leads to activation of adenylyl cyclase (AC), which in turn catalyzes the local conversion of adenosine triphosphate (ATP) into cyclic adenosine monophosphate (cAMP). The rise in cAMP then triggers protein kinase A (PKA) activation by binding to its regulatory subunits and allowing the catalytic subunit to function. PKA then acts as a nodal point and further initiates the phosphorylation of effector molecules and subsequently a functional response. Effector molecules that are phosphorylated by PKA include phospholamban, L-type calcium channels, contractile proteins, β-AR itself, as well as many others (Post et al., 1999; Rockman et al., 2002). Studies have shown that there are seven isoforms of AC found in mammalian tissue with the type 5 and 6 being the most predominant isoforms found in the heart (Post et al., 1999; Chen et al., 2012). ACs can be activated by G_αs_ signaling and deactivated by G_αi_ signaling. cAMP signaling compartmentalization is now an established concept of how this second messenger is able to differentiate between various pathways.

All three β-ARs have the ability to activate G_αs_. Therefore, there must be a system in place to differentiate the signals when the different receptors are activated. Both β_1_-AR and β_2_-AR activate G_αs_ and downstream, AC and PKA. Specific stimulation of β_1_-AR can induce cardiac hypertrophy and/or promote cardiomyocyte apoptosis (Engelhardt et al., 1999). On the other hand, under stress conditions such as in hypoxia, β_2_-AR specific stimulation activates the G_αi_ and phosphoinositol 3-kinase dependent anti-apoptotic pathway, which is not demonstrated in β_1_-AR stimulation (Chesley et al., 2000). Interestingly, Epi increases cellular cAMP levels and is able to induce activation of glycogen phosphorylase (Chen et al., 2012).

### GRKs, β-Arrestins, and Intracellular Signalosome

Termination of the signal is accomplished at various steps throughout the β-AR pathways as a balance of activation and deactivation is critical for normal cellular functioning. β-ARs themselves can be deactivated and this is mainly accomplished through the actions of GPCR kinases (GRKs). GRKs are a family of seven serine/threonine protein kinases, which canonically recognize and phosphorylate agonist-activated GPCR’s in order to terminate signaling (Sato et al., 2015). This recruits β-arrestins, which uncouple the receptor from G-proteins and promote internalization and down regulation of the receptor via a clathrin mediated process (Sato et al., 2015). Because of the sheer number of physiological processes mediated by GPCR’s, including β-ARs, GRK’s are crucial in maintaining cardiovascular homeostasis (Sato et al., 2015). Downstream of the receptors, β-arrestins can terminate signaling by recruiting phosphodiesterases (PDEs) and diacylglycerol kinase, which are involved in the breakdown of second messengers (Shenoy and Lefkowitz, 2011). β-arrestins are able to initiate signaling cascades independent of G protein activation; β_2_-AR can initiate ERK signaling both dependent on G proteins as well as independent through β-arrestins (Patel et al., 2008). PKA is also able to induce both homologous desensitization as well as β-AR agonist independent-heterologous desensitization by phosphorylation of the receptor (Post et al., 1996, 1999).

The working paradigm regarding signal differentiation involves specific subcellular localizations that are isolated from each other. These distinct intracellular signaling compartments form “signalosomes” that mediate specific responses downstream of each receptor subtype (Lohse et al., 2003). There are numerous proteins that make up these signalosomes; however, the key members that regulate these include familiar names such as AC, PKA, PDEs as well as A kinase-anchoring proteins (AKAPs). AKAPs are scaffolding proteins that assemble protein complexes including PKA and PDEs. These help to create subcellular compartments, which allows for precise spatiotemporal regulation (Tilley, 2011). PDEs break down cAMP into 5′-AMP and therefore act as another regulatory protein in maintaining tight control of local cAMP levels. In addition to AKAPs creating multiprotein complexes, physical compartmentalization of cAMP occurs via caveolae and T-tubules. Hence, understanding the complexity of β-AR signaling is fundamental for cardiac physiology and, as we will address in the following sections, for cardiac disease and physiological cardiac aging as well.

## β-Adrenergic Signaling in Heart Failure

### The Pathophysiology of Heart Failure

**FIGURE 2:**
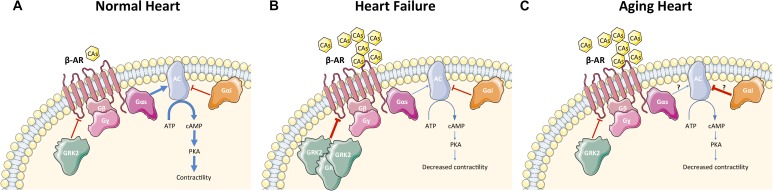
β-AR signaling in cardiomyocytes under physiological condition **(A)**, during heart failure **(B)**, and during cardiac aging **(C)**. Physiological conditions **(A)**: GRK2 phosphorylates β-ARs in cardiomyocytes and regulates contractility via AC-PKA pathway activation. Heart failure **(B)**: Increased circulating CAs led to hyper-stimulation of β-ARs and GRK2 upregulation in cardiomyocytes, resulting in desensitization/downregulation of β-ARs, ultimately leading to reduction of contractility. Aging heart **(C)**: Aging is characterized by increased circulating CAs with desensitization/downregulation of cardiomyocyte β-ARs (GRK2 is unchanged and the mechanism of β-AR dysfunction is still unknown) and decreased contractility. AC activity is reduced leading to decreased levels of cAMP. CAs, catecholamines; β-AR, β-adrenergic receptor; G-protein subunits: G_α_ (G_αs_ or G_αi_), G_β_, G_γ_; GRK2, G protein-coupled receptor kinase 2; AC, adenylyl cyclase; ATP, adenosine tri-phosphate; cAMP, cyclic adenosine mono-phosphate; PKA, protein kinase A. A blue arrow is used when a stimulatory mechanism is involved while a red bar-headed line is used for an inhibitory mechanism. Thicker arrow means upregulation while thinner arrow means downregulation.

Heart failure is a complex systemic syndrome that occurs when the heart is unable to provide tissues with adequate blood for their metabolic demands. This condition can result from alteration of systolic or diastolic function or, commonly, both. HF can be the end point of numerous CVDs such as CAD and following myocardial infarction (MI), hypertension, cardiomyopathies, valvular disorders, congenital heart diseases, rhythm abnormalities, and others. To counteract HF, it is crucial to understand the molecular mechanisms that interfere with the regulation of β-AR signaling (**Figure 2**). HF is characterized by hyperactivation of the SNS, leading to an increase in circulating CAs (NEpi and Epi) (**Figure 2**) from the adrenal medulla and augmented NEpi spillover from activated cardiac sympathetic nerve terminals into the circulation (Pepper and Lee, 1999; Lymperopoulos et al., 2013; de Lucia et al., 2014). SNS overactivity is initially a compensatory mechanism to maintain cardiac performance and stimulate contractility. Unfortunately, this chronic stimulation becomes detrimental for β-ARs and causes dysfunction in their signaling, decreases cardiac contractility, as well as diminishes inotropic reserve (Bristow et al., 1982, 1986; Rengo et al., 2009a). In addition to regulation of contractility and excitation–contraction coupling, increased CAs negatively affect LV remodeling, fibrosis, and angiogenesis (Lymperopoulos et al., 2013; Ferrara et al., 2014). Importantly, ischemia-induced SNS overdrive affects metabolism and survival of cardiomyocytes particularly if associated to other cardiovascular risk factors such as hypertension and diabetes (Parati and Esler, 2012; Dei Cas et al., 2015). At the molecular level, myocardial β-AR dysfunction in HF is characterized by loss of β_1_-AR density (around 50%) at the plasma membrane (downregulation) and by uncoupling of the membrane β_1_-ARs and β_2_-ARs from G proteins (desensitization) (Bristow et al., 1986; Ungerer et al., 1993) (**Figure 2**). When the role of β-ARs in diminished contractility in HF was first elucidated, the initial hypothesis was to increase inotropy through stimulation of cardiac β-ARs via agonists such as Epi, NEpi, and dobutamine as a clinical treatment for HF. However, the early enthusiasm diminished quickly, as patients either became hypertensive or arrhythmic after β-AR agonist therapy (Silver et al., 2002). These results, in combination with the demonstration of cardiac β-AR down-regulation in patients with HF, promoted the idea to study β-AR blockers in HF (Bristow et al., 1986; DiNicolantonio et al., 2015). β-blockers were initially used as a therapy to control HF-related tachycardia but surprisingly they were also found to significantly reduce mortality in patients with HF (Packer et al., 1996). β-blockers have been tested in numerous molecular and clinical studies, and they currently represent one of the cornerstones in the therapy of HF, though it is still debated whether they act by blocking or by resensitizing the β-AR system (Vinge et al., 2008).

### The Role of GRK2 in Heart Failure

Importantly, it has been clearly shown both in humans and animal models that β-AR dysfunction in HF is GRK2-mediated: SNS hyperactivity triggers GRK2 upregulation that is activated at the beginning to counteract the excessive catecholaminergic drive but later, elevated GRK2 levels leads to dysfunctional β-AR signaling and decreased contractility/inotropic reserve (**Figure 2**) (Post et al., 1999; Floras, 2002; Penela et al., 2006; Agüero et al., 2012; Rengo et al., 2012b; Sato et al., 2015). Hence, GRK2 inhibition has been proposed as a potential therapeutic target for HF (Lymperopoulos et al., 2012). Studies in a mouse model expressing the βARKct as a peptide inhibitor of GRK2 has confirmed this hypothesis. Particularly, transgenic mice that expressed βARKct showed enhanced cardiac contractility and increased sensitivity to acute β-AR stimulation (Koch et al., 1995). Conversely, cardiomyocyte-restricted overexpression of GRK2 at levels found in failing human myocardium (around threefold to fourfold) significantly decreased β-AR signaling and contractile reserve (Koch et al., 1995). Mice with cardiac βARKct expression have also been shown to reverse ventricular dysfunction in several mouse models of HF (Freeman et al., 2001; Harding et al., 2001). In addition, *in vivo* inhibition of GRK2 only in cardiomyocytes, at or after birth, was able to ameliorate cardiac contractility and reverse adverse ventricular remodeling through improved β-AR signaling in an ischemic model of HF, confirming the specific pathological role of GRK2 in cardiomyocytes (Raake et al., 2008). Studies in rats, rabbit and large animals have further endorsed the potential applicability of GRK2 inhibition in clinical practice using viral-mediated βARKct gene therapy (White et al., 2000; Shah et al., 2001; Rengo et al., 2009b, 2012b). This includes pre-clinical studies using adeno-associated virus-serotype-6 (AAV6)-mediated expression of βARKct in a porcine model of post-ischemic HF (Raake et al., 2013). In addition, paroxetine, a selective serotonin reuptake inhibitor (SSRI) that is currently used to treat depression and neuropsychiatric disorders, is also able to inhibit GRK2 as an off-target in the μM affinity range (Thal et al., 2012). HF mice chronically treated with this drug have been shown to improve left ventricular (LV) ejection fraction (EF) and remodeling after MI. Our data showed that paroxetine reversed HF in mice due to its GRK2 inhibitory actions and were not related to its SSRI activity (Thal et al., 2012; Schumacher et al., 2015). Although paroxetine may not be a perfect candidate for HF therapy due to its well known mainly neurologic consequences, its beneficial effects in heart disease have stimulated medicinal chemists to develop selective small pharmacological agents that inhibits GRK2 (Powell et al., 2016; Waldschmidt et al., 2017).

Intriguingly, several molecules have been described to have GRK2 inhibitory properties such as RNA-aptamers or molecules that target the GRK2-G_βγ_ protein-protein interaction (M119 and Gallein) (Cannavo et al., 2013). Hence, GRK2 inhibition for the treatment of HF is not far from application in clinical trials: gene therapy with AAV6-βARKct will occur in the near future while GRK2 inhibition by small molecules would be an intriguing option, as well. β-ARKct expression and small molecules are known to target G_βγ_ binding, however, the main goal of cardiac GRK2 inhibition is to prevent phosphorylation-mediated β-AR dysfunction, and ultimately restore cardiac function (Sato et al., 2015). Inhibiting GRK2 to improve β-AR signaling in HF or aging appears counter-intuitive since β-blockers are effective in HF, however, GRK2 inhibition chronically up-regulates receptors in addition to resensitizing them, restoring normal β-AR contractile and metabolic signaling that essentially reestablishes the fight-or-flight response and studies have shown this to be true with βARKct and paroxetine lowering Ca^2+^ levels and SNS over-activity in HF models (Rengo et al., 2009b; Raake et al., 2013; Schumacher et al., 2015). This is actually part of the mechanism of β-blockers as well since they up-regulate receptors and improve signaling once activated (Sato et al., 2015). Studies with GRK2 inhibition coupled with a β-blocker in HF as shown in most models to be additive or even synergistic, also supporting non-β-AR mechanisms involved in the therapeutic responses to GRK2 inhibitors (Sato et al., 2015). Moreover, our group has shown that GRK2 inhibition improved myocardial insulin signaling and cardiac metabolism in a model of ischemic HF (Ciccarelli et al., 2011).

### β-Adrenergic Receptors in Heart Failure

Recently, our lab has shown that long-term caloric restriction normalized cardiac β_1_-AR levels and improved cardiac inotropic reserve and sympathetic innervation in an animal model of ischemic HF (de Lucia et al., 2018a). Since it has been shown that β_1_-AR downregulation is detrimental in HF, Engelhardt et al. (1999) studied if β_1_-AR overexpression is positively affecting cardiac function. The authors showed that myocardial specific β_1_-AR Transgenic (Tg) mice had increased cardiac contractility at a young age but after a few months developed marked myocyte hypertrophy and heart dysfunction (reduced EF). Moreover aged β_1_-AR Tg mice showed increased levels of interstitial collagen type-I/III and augmented levels/activity of matrix metalloproteinase-2 associated with severe fibrosis (Seeland et al., 2007). Importantly, it is still debated if cardiac β_2_-AR overexpression is beneficial in HF. Several authors have shown that β_2_-AR overexpression is able to ameliorate cardiac function in cardiac hypertrophy and HF (Dorn et al., 1999; Tevaearai et al., 2002; Rengo et al., 2012c). In fact, some authors showed that myocardial β_2_-AR overexpression via gene delivery not only improved cardiac contractility and maladaptive remodeling but also increased angiogenesis in an animal model of HF (Rengo et al., 2012c). Other investigators demonstrated that cardiac β_2_-AR overexpression led to cardiac dysfunction, interstitial fibrosis, spontaneous onset of ventricular tachyarrhythmias (becoming more severe with aging), and higher mortality (Du et al., 2000; Nguyen et al., 2015). This latter result could be related to high β_2_-AR levels (300-fold overexpression) and taken together with the data of Dorn et al. (1999), suggest that β_2_-AR overexpression is probably beneficial only at a low-moderate level (Du et al., 2000). Interestingly, like many GPCRs, β_1_-AR and β_2_-AR can transactivate receptor tyrosine kinases such as epidermal growth factor receptor (EGFR). In particular, β-arrestins mediate β_1_-AR signaling to the EGFR and this mechanism is independent of G protein activation but involves GRK5 and GRK6 (Noma et al., 2007). In mice undergoing chronic sympathetic stimulation, this β-arrestin-biased signaling is shown to have a cardioprotective role, counteracting the effects of CAs toxicity. This research suggest that drugs that can antagonize G protein-mediated signaling and at the same time stimulate the β-arrestin-mediated cardioprotective pathway, would be useful in pathologies where SNS overactivity is prominent, such as HF or diabetes-related cardiac disease (Noma et al., 2007; Iyngkaran et al., 2013; Lymperopoulos et al., 2013; Rengo et al., 2015). Besides, β_1_-AR and β_2_-AR, the β_3_-AR is found in cardiac myocytes with a characteristic coupling (G_αs_ and G_αi_) and effects on cardiac function and remodeling (Moniotte et al., 2001; Balligand, 2013). In particular, β_3_-AR is upregulated in cardiac diseases (both in human and animal models) and is less sensitive to desensitization compared to β_1_-AR and β_2_-AR (Dinçer et al., 2001; Moniotte et al., 2001; Balligand, 2013). Several groups have examined the effect of β_3_-AR agonists *in vivo* in healthy animals or models of HF, with conflicting results. Some authors argue that β_3_-AR agonists have a positive inotropic effect (mainly in HF models) (Donckier et al., 2001; Morimoto et al., 2004; Bundgaard et al., 2010; Balligand, 2016). Particularly, one of these studies found only a small negative inotropic effect in healthy sheep, but a significant improvement of inotropic parameters in sheep with HF. The latter *in vivo* effect was consistent with a beneficial effect of Na^+^-K^+^ ATPase stimulation (Bundgaard et al., 2010). These different results from different groups, were possibly due to the type of β_3_-AR agonist (diverse β_3_-AR specificity) and dose of agonist used (high dose would potentially induce opposite effects compared to the low dose) (Balligand, 2016). Intriguingly, β_3_-AR in cardiomyocytes, via-paracrine stimulation, influences surrounding cells (e.g., fibroblasts and endothelial cells). In particular, β_3_-AR overexpression in myocytes reduced myocardial interstitial fibrosis and increased angiogenesis in response to isoproterenol/angiotensin II infusions as well as a β_3_-AR agonist in mice submitted to transaortic constriction model (Niu et al., 2012; Belge et al., 2014; Balligand, 2016). Nevertheless, despite interesting results in basic research, Mirabegron (a β_3_-AR agonist) did not increase EF or affect LV volumes in HF patients (results from the BEAT-HF trial) (Bundgaard et al., 2017). However, the authors did an exploratory sub-analysis showing an increase in EF in patients with severe HF at baseline, but not in patients with EF ≥ 40%. This analysis has been based on the hypothesis that β_3_-AR agonist-induced Na^+^-K^+^ ATPase stimulation is more effective in severe HF. Additional studies on the effects of β_3_-AR agonists in patients with severe HF will clarify these results.

Interestingly, our lab has recently showed that β_3_-AR dysfunction may be involved in patients that do not respond to β_1_-AR-blockers (Cannavo et al., 2017). In fact, Metoprolol does not improve cardiac function (EF) and fibrosis in mice with β_3_-AR deletion post-MI (Cannavo et al., 2017). β_1_-AR blockade by Metoprolol leads to up-regulation of β_3_-ARs, followed by the activation of sphingosine-1-phosphate (S1P) receptor signaling that appears protective and beneficial (Cannavo et al., 2017).

### β-Arrestins, Adenylyl Cyclases, and AKAPs in Heart Failure

Another important component of β-AR signaling are β-arrestins (see above) and our group has studied their role in HF. Particularly, β-arrestin 1 knockout (KO) mice have shown improved cardiac function (enhanced EF and inotropic reserve) in a model of ischemic HF: the underlying mechanisms are, on one side, improved cardiac β-AR signaling and function due to cardiac β-arrestin 1 deletion, and on the other, decreased circulating levels of CAs and aldosterone due to adrenal β-arrestin 1 absence (Lymperopoulos et al., 2011; Bathgate-Siryk et al., 2014). Contrasting data have been published on the effects of β-arrestin 2 manipulation during ischemic injury. β-arrestin 2 KO mice showed lower survival compared to WT mice after MI due to enhanced macrophages-induced cardiac inflammation (Watari et al., 2013). In addition, β-arrestin 2 overexpression improved cardiac contractility and LV remodeling after MI through increased sarco[endo]plasmic reticulum Ca^2+^-ATPase (SERCA2a) activity (McCrink et al., 2017). On the other hand, Wang et al. (2017) showed that β-arrestin 2 KO mice are resistant to myocardial damage caused in an ischemia/reperfusion injury model. These dissimilar results on the role of β-arrestin 2 may be due to the different injury model used and related pathways involved. In this regard, further studies are necessary to understand if β-arrestin 2 deletion or overexpression is beneficial in ischemic HF.

Furthermore, it has been hypothesized that interrupting distal mechanisms in the β-AR-G protein-AC pathway may also be a therapeutic target in HF (Ho et al., 2010). Deletion of AC type 5 improves survival and protects against chronic pressure overload, chronic β-AR stimulation or enhanced β-AR signaling (mice with overexpressed β_2_-AR) via-anti apoptotic and anti-oxidative mechanisms (Okumura et al., 2003, 2007; Yan et al., 2014). In addition, activation of cardiac AC type 6 in mice with ischemic injury, improved LV systolic and diastolic function (enhanced EF and increased slope of the end systolic pressure-volume relationship – ESPVR), and decreases apoptosis (Lai et al., 2008). These studies suggest that AC type 5 inhibition or AC type 6 activation would be interesting therapeutic approaches in HF.

Mitochondrial AKAPs (mainly AKAP1) have been demonstrated to influence mitochondrial function and reactive oxygen species production in the heart. AKAP1 deletion led to mitochondrial alterations, enhanced mitophagy and increased infarct size as well as LV pathological remodeling in a model of post-ischemic HF (Schiattarella et al., 2016). In addition, AKAP5 deletion caused cardiac dilatation and dysfunction via enhanced activity of calmodulin kinase II/calcineurin and altered recycling of cardiac β_1_-ARs (Li et al., 2014). Thus, AKAPs might represent another important player in cardiac pathophysiology.

### Cross-Talk Between Beta-Adrenergic and Insulin Signaling in Heart Failure

It has also been reported that the prevalence of diabetes is augmented in patients with HF and hyperglycemia/hyperinsulinemia can strongly contribute to the impairment of cardiac function as well as worsen prognosis (Dei Cas et al., 2015; Jia et al., 2016; Komici et al., 2018). Furthermore, an insulin-resistant state (insulin resistance in both cardiac and peripheral tissues) seems to occur as a consequence of increased circulating CAs, in HF patients (Paolisso et al., 1991; Shimizu et al., 2012; Fu et al., 2017). In this regard, it is not surprising that both HF and diabetic patients show SNS overactivity (Lymperopoulos et al., 2013; Han et al., 2016). The insulin receptor (Ins-R) and β-AR cross talk, share similar down-stream signaling components, such as G_αi_, β-arrestins and GRK2, and counter-regulate each other (Song et al., 2001; Morisco et al., 2005; Cipolletta et al., 2009; Garcia-Guerra et al., 2010; Mangmool et al., 2016; Fu et al., 2017). This strict interaction was further clarified when a direct interaction in a membrane complex was found between Ins-R and β_2_-AR (Mandić et al., 2014). Acute cardiac β-AR activation potentiates insulin-induced Akt-mediated glucose uptake via PKA, while chronic β-AR stimulation leads to insulin resistance (characterized by sustained Akt activation and reduced Glucose transporter type 4 -GLUT4- membrane levels) and ultimately impaired glucose uptake (Morisco et al., 2005; Mangmool et al., 2016; Fu et al., 2017). On the other hand, it has been shown that chronic insulin stimulation directly impairs cardiac β-AR signaling in mice (Fu et al., 2014, 2015). Interestingly, insulin elicits GRK2 recruitment to the plasma membrane and following β_2_-AR phosphorylation/internalization, eventually reduces cAMP-PKA activity and contractile response in cardiomyocytes (Fu et al., 2015). Insulin receptor substrate 2 (IRS2) is crucial for the interaction between GRK2 and Ins-R (Fu et al., 2015). In addition, insulin stimulation induces PKA phosphorylation of the β_2_-AR as well as β_2_-AR coupling to G_αi_ (Fu et al., 2014). GRK2 is emerging as a key link to connect cardiac insulin and adrenergic signaling, as this kinase on one side can be upregulated by CAs and/or hyperinsulinemia and, on the other, phosphorylates and desensitizes both β-AR and Ins-R (Ciccarelli et al., 2011; Fu et al., 2015, 2017; Mayor et al., 2018). In addition, cardiac GRK2 levels increase, not only during HF, but also in insulin resistance status such as in ob/ob mice or animals fed with high fat diet (Lucas et al., 2014). Our group has shown that GRK2 impairs cardiac glucose uptake and promotes insulin resistance after myocardial ischemia in an animal model, confirming that GRK2 could be an important hub in the regulation of cardiac function and metabolism during cardiac stress (Ciccarelli et al., 2011; Woodall et al., 2014; Mayor et al., 2018). Further, GRK2 downregulation enhances basal cardiac insulin sensitivity and ameliorates insulin resistance in an animal model of high-fat diet-induced obesity (Lucas et al., 2014; Vila-Bedmar et al., 2015). GRK2 is also involved in insulin signaling/IR dysfunction in other tissue as its levels are increased in muscle and adipose tissue in the animal models of insulin resistance as well as in lymphocytes from patients with metabolic syndrome (Garcia-Guerra et al., 2010). Moreover, GRK2 KO mice displayed protection and enhanced insulin sensitivity in animal models of obesity and TNFα-induced insulin resistance (Garcia-Guerra et al., 2010). Diabetic patients are more likely to have infections and develop sepsis (Schuetz et al., 2011). GRK2 plays an important role in the pathogenesis and outcomes of sepsis in both animal models and patients by regulating inflammation and immune system (Packiriswamy and Parameswaran, 2015). The role of GRK2 as crucial node between HF and diabetes has been confirmed in humans as well. In fact, lymphocyte GRK2 protein levels are significantly increased in patients with diabetes mellitus and HF compared to failing non-diabetics patients, suggesting further compromised cardiac β-AR signaling/function (Rengo et al., 2015). Of note, myocardial GRK2 expression and activity are mirrored by lymphocyte levels of this kinase and consequently, lymphocyte GRK2 may be an adequate surrogate for monitoring cardiac GRK2 in human HF (Iaccarino et al., 2005). Incretin mimetics and inhibitors of the protease dipeptidyl peptidase-4 (DPP-4) are new promising classes of anti-diabetic agents that improve insulin sensitivity and pancreatic beta-cell function (Namba et al., 2013; Tasyurek et al., 2014). Interestingly, DPP-4 inhibition reduced isoproterenol-induced cardiomyocyte hypertrophy and perivascular fibrosis ameliorating cardiac glucose metabolism and inflammation in rats (Miyoshi et al., 2014).

## β-Adrenergic Signaling in Aging Heart

### The Pathophysiology of Aging Heart

Cardiac aging in healthy individuals is characterized by numerous pathophysiological changes affecting the heart at the structural, histological, molecular and functional level. Structural changes primarily include LV remodeling, valvular changes, and modification in the conduction system while histologic changes involve fibrosis and modifications of extracellular matrix as well as cardiomyocyte death and hypertrophy (Boyle et al., 2011; Dai et al., 2012; Ferrara et al., 2014). At the molecular level cardiac aging shows altered excitation–contraction coupling and increased oxidative stress. The aforementioned alterations are closely recapitulated in animal models used in aging studies (Chiao and Rabinovitch, 2015). Functional alterations typically involve diastolic function while age-related systolic dysfunction is still controversial (Strait and Lakatta, 2012; de Lucia et al., 2018b). It has recently been shown in both healthy patients and aging animal model that systolic function evaluated with speckle-tracking analysis was impaired (Crendal et al., 2014; de Lucia et al., 2018b). Importantly, in the senescent heart, sympathetic activity is increased while cardiac neuronal uptake of CAs is decreased (Tsuchimochi et al., 1995; Ferrara et al., 2014). Age-dependent β-AR dysfunction is characterized by reduction in receptor density (White et al., 1994; Cerbai et al., 1995; Ferrara et al., 2014). β-adrenergic desensitization during aging causes reduced exercise tolerance, altered LV inotropic reserve, arterial-ventricular load mismatching, and physical deconditioning (Davies et al., 1996; Ferrara et al., 2014). In fact, the effects of aging are most evident during an exercise stress test which shows an overall decline in VO_2_max and cardiac index (Leosco et al., 2013). In addition, older healthy patients have a lower increase in heart rate during exercise and are less susceptible to β-blockade compared to young people (Ferrara et al., 2014; Nakou et al., 2016).

### Molecular Mechanisms in Cardiac Aging: Role of β-Adrenergic Signaling

Although changes in β_1_- and β_2_-AR agonist response in the failing and aging human heart are quite similar, GRKs expression and activity seem to be unaffected by age (Xiao et al., 1998; Leineweber et al., 2003) (**Figure 2**). Hence, the main cellular mechanism for reduced cardiac β-AR responsiveness during aging is unknown. It is hypothesized that there is another mechanism other than GRKs that determines dysfunctional β-AR signaling during the aging process. Importantly, contrasting results have been published about cardiac G_αi_ levels content and activity. In one study, G_αi_ levels were measured in atrial tissues received from cardio-surgical patients and it was found that G_αi_ expression increased with age (Brodde et al., 1995a,b). Age-dependent G_αi_ upregulation has been shown in animal models (rat), as well (Böhm et al., 1993). Oppositely, Xiao et al. (1998) reported that G_αi_ activity is unchanged in aged hearts (rat model) and probably do not contribute to the age-related reduction in cardiac β-AR dysfunction. Interestingly, another group found that G_αi_ content is unchanged while G_αi_ activity is increased in a Guinea-pig aging model (Ferrara et al., 1997a,b). These dissimilar results, most likely, are due to the use of different animal species or time-points (early aging or late aging). Aging is associated with decreased AC activity and cAMP production in the heart (Narayanan and Tucker, 1986; Tang et al., 2011). Cardiac overexpression of AC type 6 ameliorates age-related cardiac dysfunction (increased EF and slope of ESPVR), as showed in HF (see above), through improved calcium uptake (Tang et al., 2011).

Taken together, these studies suggest that β-AR dysfunction is an important determinant in the age-related cardiac alterations and understanding the specific mechanisms involved would be beneficial for designing specific treatments for the elderly patients with cardiac dysfunction.

## Clinical Point of View and Future Directions

### The Intersection Between Heart Failure and Cardiac Aging

Over the last few decades, life expectancy has significantly increased although several diseases persist with aging as a risk factor. Particularly, despite the improvement in treatments, many elderly people suffer from cardiac problems (HF, valvular diseases, arrhythmias or hypertension) that are much more common in an older fragile heart (Nakou et al., 2016). Interestingly, during both HF and aging, the heart may increase in size and cannot effectively pump blood to the other organs. Cardiac dysfunction is mainly diastolic during aging while it is commonly both systolic and diastolic in HF (different causes could determine the prevalence of one over the other) (Hees et al., 2002; Khouri et al., 2005; Najafi et al., 2016). Nevertheless, there are many similarities between the aging and failing heart: histologically, both have myocyte hypertrophy and cardiac fibrosis and functionally show decreased inotropic reserve and resistance to exercise training (Najafi et al., 2016). In the long-term, age-related heart dysfunction and HF together contribute to the decrease in wellness and performance of normal daily activities for elderly people (De Geest et al., 2004; Kalogeropoulos et al., 2009; Goldwater and Pinney, 2015; Uchmanowicz et al., 2015). In the last decade, epidemiological studies have shown a high incidence and prevalence of HF in the elderly, mainly due to increased life expectancy and improvement in the treatment of MI and its complications. About 50% of all HF cases are found in people older than 70 years (Ho et al., 1993; Mozaffarian et al., 2016). The outcomes in elderly patients with HF are modest: older people are seen in a more advanced stage of HF (NYHA class III-IV) and have a 50% survival rate after a 4-year period (Cacciatore et al., 1998; Pulignano et al., 2002).

### β-Adrenergic Signaling as Therapeutic Target and Diagnostic Tool in Heart Disease

Many studies have linked the dysfunction of the β-AR system with the pathogenesis of both HF and senile heart (De Geest et al., 2004). In both conditions, there is an increase in circulating CAs as a result of their reduced plasma clearance, augmented adrenal production, or augmented spillover from the tissues. Moreover, there is an age-dependent and HF-related reduction of the CA re-uptake transporter localized in the sympathetic nerve terminals in the heart (Leineweber et al., 2002; Leosco et al., 2015; Rengo et al., 2016b). CA levels are significantly correlated with survival and symptoms in patients with HF (Cohn et al., 1984; Grassi et al., 2009). The cardiac β-AR system is desensitized in aging and the failing heart and G_αi_ activity appears to be altered in both conditions, as well. Although β-AR dysfunction in HF is primarily related to GRK2 upregulation, the mechanism involved in age-related β-AR altered levels/function is still unclear (Xiao et al., 1998). Possibly, GRK2 upregulation in HF is triggered by a stressor event such as MI that prompts acute and robust CA increase while SNS overdrive during aging is gradual and occurs over many years. The importance of β-ARs in the heart has become greater since many treatments that restore their function and signaling has been demonstrated to be beneficial in the treatment of HF and age-related heart dysfunction (Leosco et al., 2007, 2008, 2013; Rengo et al., 2013). In fact, Leosco et al. (2007, 2008) proved that both physical activity and metoprolol, alone or in combination, improved β-AR signaling in the aged heart, suggesting a similar effect on β-AR signaling of chronic treatment with β-blockers and exercise training. Exercise and β-blockers have been shown to resensitize cardiac β-ARs via modulation of cardiac GRK2 levels/activity and G-protein-dependent AC activation (Leosco et al., 2007). In addition, both treatments can downregulate the HF-related SNS overactivity, attenuating adrenal GRK2 overexpression, increasing adrenal α_2_ adrenergic receptor density, and finally reducing CA secretion from the adrenal medulla (Rengo et al., 2010, 2012a, 2013; Femminella et al., 2013). Moreover, physical activity can counteract age-dependent cAMP decline via decreased expression of G_αi_ and enhanced isoprenaline-stimulated AC activity (Böhm et al., 1993). Another approach for HF would be to inhibit cardiac GRK2 in order to restore β-AR downregulation/desensitization. In this regard, GRK2 inhibition via gene therapy is not far from application in a clinical trial. However, our group has recently shown that paroxetine, currently used as SSRI, is a GRK2 inhibitor (Thal et al., 2012; Schumacher et al., 2015; Powell et al., 2016). This latter discovery is encouraging pharmacologists to develop selective small molecules that are able to inhibit GRK2 (Waldschmidt et al., 2016). It has been found that an inverse correlation exists between β-ARs and GRK2 levels. Intriguingly, cardiac GRK2 is associated to the degree of LV dysfunction, suggesting this kinase can act as a HF progression biomarker (Sato et al., 2015). Moreover, Rengo et al. (2016a) showed that lymphocyte GRK2 protein levels can independently predict prognosis in patients with HF. In fact, lymphocyte GRK2 levels showed an additional prognostic and clinical value over demographic and clinical variables (Rengo et al., 2016a). The same group performed a prospective study in a group of HF patients who underwent rehabilitative treatment through physical activity. Exercise was associated with a significant reduction of lymphocyte GRK2 protein levels and HF patients who did not demonstrate reduced lymphocyte GRK2 protein levels after training presented with a worse outcome (Rengo et al., 2014). Hence, lymphocyte GRK2 may be considered as a promising prognostic marker in HF patients (Travers et al., 2016).

MicroRNAs (miRNAs) are a class of small non-coding RNAs that regulate gene expression during cardiac aging and age-related CVDs (Kondkar and Abu -Amero, 2015; de Lucia et al., 2017). miRNAs are emerging as a stimulating therapeutic target and have been shown to be stable biomarkers for diagnosis and prognosis during cardiac dysfunction (Kondkar and Abu -Amero, 2015). miR-133 has been recently shown to regulate several components of the β_1_-AR transduction signaling and is cardio-protective during pressure overload-induced HF (via decreased apoptosis and fibrosis) (Castaldi et al., 2014).

Despite the increasing effort in basic research on the age-related cardiac dysfunction, clinical research is deeply underestimating the need for studies that include elderly patients. In fact, there is a huge disparity between research and market demand: patients ≥65 are under-represented in all phases of clinical trials, even though in HF, these patients are the primary drug users. In this regard, it would be important to invest (in basic and importantly then in clinical research) in development of treatments that will restore β-AR signaling in both HF and aging heart.

## Conclusion

The aged population is drastically increasing worldwide and it is mandatory to assure an adequate quality life to the elderly suffering from decreased cardiac function. In fact, age-related cardiac alterations and HF, deeply influence the autonomy and the ability to perform daily activities for elderly people. Importantly, HF is one of the most important comorbidities and causes of hospitalization and/or death for old people in western countries, dramatically affecting the cost of healthcare. Hence, there is a great need for treatments that ameliorate cardiac dysfunction in older people acting against both HF and cardiac physiological aging. Since β-AR signaling is dysfunctional in failing as well as aging heart, this pathway is an effective diagnostic and therapeutic target.

## Author Contributions

All authors participated in writing the manuscript and/or revising it critically for important intellectual content.

## Conflict of Interest Statement

The authors declare that the research was conducted in the absence of any commercial or financial relationships that could be construed as a potential conflict of interest.
